# Domestic Violence Housing First Model and Association With Survivors’ Housing Stability, Safety, and Well-being Over 2 Years

**DOI:** 10.1001/jamanetworkopen.2023.20213

**Published:** 2023-06-26

**Authors:** Cris M. Sullivan, Cortney Simmons, Mayra Guerrero, Adam Farero, Gabriela López-Zerón, Oyesola Oluwafunmilayo Ayeni, Danielle Chiaramonte, Mackenzie Sprecher, Aileen I. Fernandez

**Affiliations:** 1 Department of Psychology, Michigan State University, East Lansing; 2 Department of Psychology, Yale University, New Haven, Connecticut; 3 Department of Psychology, University of Illinois, Chicago; 4 Department of Psychology, University of Michigan, Ann Arbor; 5 National Resource Center on Domestic Violence, Harrisburg, Pennsylvania; 6 Department of Social and Behavioral Sciences, Yale University, New Haven, Connecticut; 7 School of Social Work, Wayne State University, Detroit, Michigan

## Abstract

**Importance:**

Intimate partner violence (IPV) is a leading cause of homelessness and a serious threat to public health and well-being.

**Objective:**

To determine whether the Domestic Violence Housing First (DVHF) model improves safety, housing stability, and mental health over 2 years.

**Design, Setting, and Participants:**

This longitudinal comparative effectiveness study interviewed IPV survivors and reviewed their agency records. All unstably housed or homeless IPV survivors entering domestic violence (DV) services were eligible to participate in the study, ensuring capture of typical variability in service delivery (eg, some survivors would enter services when agencies had the capacity to provide DVHF and others would receive services as usual [SAU]). Clients from 5 DV agencies (3 rural and 2 urban) referred by agency staff in a Pacific Northwest state of the United States were assessed between July 17, 2017, and July 16, 2021. Interviews were conducted in English or Spanish at entry into services (baseline) and at 6-, 12-, 18-, and 24-month follow-up visits. The DVHF model was compared with SAU. The baseline sample included 406 survivors (92.7% of 438 eligible). Of the 375 participants retained at the 6-month follow-up (92.4% retention), 344 had received services and had complete data across all outcomes. Three hundred sixty-three participants (89.4%) were retained at the 24-month follow-up.

**Intervention:**

The DVHF model has 2 components: housing-inclusive advocacy and flexible funding.

**Main Outcomes and Measures:**

Main outcomes included housing stability, safety, and mental health, which were assessed using standardized measures.

**Results:**

Of the 344 participants (mean [SD] age, 34.6 [9.0] years) included in the analyses, 219 (63.7%) received DVHF and 125 (36.3%) received SAU. Most participants identified as female (334 [97.1%]) and heterosexual (299 [86.9%]). Two hundred twenty-one participants (64.2%) were from a racial and ethnic minority group. Longitudinal linear mixed-effects models showed that receiving SAU was associated with greater housing instability (mean difference, 0.78 [95% CI, 0.42-1.14]), DV exposure (mean difference, 0.15 [95% CI, 0.05-0.26]), depression (mean difference, 1.35 [95% CI, 0.27-2.43]), anxiety (mean difference, 1.15 [95% CI, 0.11-2.19]), and posttraumatic stress disorder (mean difference, 0.54 [95% CI, 0.04-1.04]) compared with receiving the DVHF model.

**Conclusions and Relevance:**

Evidence in this comparative effectiveness study suggests that the DVHF model was more effective than SAU in improving the housing stability, safety, and mental health of survivors of IPV. The DVHF’s amelioration of all of these interconnected public health issues—relatively quickly and with long-term continuance—will be of substantial interest to DV agencies and others working to support unstably housed IPV survivors.

## Key Points

**Question:**

Is the Domestic Violence Housing First (DVHF) model associated with safety, housing stability, and reduced mental health symptoms over 24 months?

**Findings:**

In this comparative effectiveness study of 344 survivors of intimate partner violence, receiving the DVHF model was associated with greater safety and housing stability and with reduced mental health symptoms over 24 months compared with receiving services as usual.

**Meaning:**

This study provides evidence of the effectiveness of DVHF in promoting the safety and well-being of survivors over time, highlighting the importance of investing in domestic violence agencies’ use of survivor-driven housing advocacy and flexible funding.

## Teaser

This comparative effectiveness study assesses whether survivors of intimate partner violence demonstrate greater improvements in housing stability, safety, and mental health after receiving an intervention including the Domestic Violence Housing First model compared with services as usual across 2 years.

## Introduction

Intimate partner violence (IPV) is a significant public health concern worldwide,^1^ disproportionately affecting women.^2^ Intimate partner violence includes violence, abuse, and controlling behavior committed by a partner or former partner^3^ and can result in long-lasting adverse mental health outcomes, including depression,^4^ posttraumatic stress disorder (PTSD),^5^ and anxiety.^6^ Intimate partner violence is also a leading cause of homelessness and housing instability.^7,8,9^

Given the link between IPV and housing instability, as well as the lack of affordable housing in the US,^10^ domestic violence (DV) agencies are increasingly focusing on helping IPV survivors achieve stable housing along with long-term safety and well-being.^11,12^ One intervention that is increasingly being implemented by DV agencies is the Domestic Violence Housing First (DVHF) model. Adapted from the Housing First model, which was originally developed for chronically homeless, single adults struggling with mental health disorders and addictions,^13^ the DVHF model includes 2 components: survivor-driven housing-inclusive advocacy and flexible funding.^11^ The former component involves advocates working proactively and creatively with survivors to help them obtain safe and stable housing, at their own pace, for as long as the survivor needs support. Flexible funding involves assisting survivors with individual needs that are preventing them from achieving safe and stable housing. Funding is used to help cover a myriad of expenses, including but not limited to rent, car repair, employment-related expenses, moving expenses, and safety measures. The model was created based on evidence from 2 earlier studies: one demonstrating the effectiveness of survivor-driven advocacy on IPV survivors’ safety and mental health,^14^ and one showing that flexible funding can reduce IPV survivors’ housing instability.^15^

Given that the DVHF model continues to proliferate, empirical evidence is needed to understand the extent of its effectiveness. This study tested the hypothesis that IPV survivors who received the DVHF model would demonstrate greater improvements in housing stability, safety, and mental health compared with survivors who received services as usual (SAU) across 2 years.

## Methods

### Procedure

We intentionally chose a nonrandomized comparative effectiveness design for this study to capitalize on how services are provided in community settings.^16^ Due to staff turnover, fluctuation in agency resources, and other factors typical of nonprofit human service agencies, we knew that some survivors would receive DVHF while others would receive SAU, such as support groups, counseling, legal advocacy, and referrals.^17^ Written informed consent was obtained from participants before the start of the study. Institutional review board research approval was obtained from Michigan State University. This report follows the International Society for Pharmacoeconomics and Outcomes Research (ISPOR) reporting guidelines for nonrandomized comparative effectiveness research.

Participants were recruited from 5 DV survivor service organizations (2 urban and 3 rural) in Washington State between July 17, 2017, and July 16, 2021. Agency staff referred all homeless or unstably housed clients to the study, and the final sample included 406 survivors (92.7% of 438 eligible). Interviews were conducted in English or Spanish, and participants were paid $50 for each interview. Interviews were conducted either in-person or over the telephone (based on participant preference), and participants were interviewed 5 times over 24 months (at baseline and 6, 12, 18, and 24 months). Interviews included questions about housing instability, abuse, mental health, and services received. The baseline interview also captured basic self-reported demographic information as well as historical data regarding abuse and homelessness. Agency records provided information about flexible funding and services received.

### Study Variables

Housing instability was measured with the 7-item Housing Instability Scale.^18^ Of the 7 scale items, 5 included dichotomous yes or no responses, and 2 items were recoded to be dichotomous. Scores ranged from 0 to 7, with higher scores indicating higher instability. Cronbach α was examined at each wave of data collection, with overall α = 0.79. The Housing Instability Scale has established predictive and concurrent validity across English and Spanish.

Physical, emotional, and sexual abuse, as well as stalking and harassment, were measured using a 31-item scale created by modifying the Composite Abuse Scale (CAS).^19^ Validation studies have found the CAS to have high internal consistency.^20^ Two CAS items (“hang around outside your house” and “harass you at work”) were replaced with “repeatedly follow you, phone you, and/or show up at your house/work/other place” to capture multiple indicators of stalking that were relevant even if the participant was living with the abuser. Four items were added to address abusive behaviors not adequately measured in the original scale: (1) stalk you, (2) strangle you, (3) demand sex whether you wanted to or not, and (4) force sexual activity. Responses were recorded using a 6-point scale ranging from 0 indicating never to 5 indicating daily, with higher scores indicating greater abuse (Cronbach α = 0.94).

Depression was measured by the 9-item Patient Health Questionnaire.^21^ Responses for each item were recorded using a 4-point scale ranging from 0 indicating not at all to 3 indicating nearly every day, referring to the prior 2 weeks. Scores ranged between 0 and 27, with higher scores indicating higher depression (Cronbach α = 0.88).

Anxiety was assessed using the 7-item Generalized Anxiety Disorder measure.^22^ Responses for each item were recorded using a 4-point scale ranging from 0 indicating not at all to 3 indicating nearly every day. Scores ranged between 0 and 21, with higher scores indicating higher anxiety (Cronbach α = 0.91).

Posttraumatic stress disorder was assessed using the 10-item Trauma Screening Questionnaire.^23^ Participants responded to items regarding physical and emotional responses to trauma and indicated yes or no (0 indicating no and 1 indicating yes) if they experienced any of the symptoms at least twice in the prior week. Scores could range from 0 to 10, with higher scores indicating a higher degree of PTSD (Cronbach α = 0.75).

### Statistical Analysis

Several procedures were implemented before hypothesis testing to account for potential bias in the sample that could affect findings. Inverse probability weights (IPWs)^24^ were calculated and included in the longitudinal models as sampling weights to account for any selection bias resulting from factors that increased the probability that certain individuals received the intervention (eMethods in Supplement 1). To compute the IPWs, we first used logistic regression models to examine whether there were any meaningful baseline differences between those who received DVHF vs those who received SAU across 72 variables (eTable 1 in Supplement 1). Statistical significance was set at 2-sided *P* < .05. Thirteen factors were identified and used to calculate the IPWs: parenting children (yes or no), living with the abuser (yes or no), racial or ethnic minority group (yes or no), having been in foster care as a child (yes or no), housing barriers, housing instability, staying with friends to avoid homelessness (yes or no), inability to make ends meet, overall abuse, alcohol misuse, drug misuse, quality of life, and whether or not the DV agency was in a rural area (yes or no). The results of the diagnostics used to ensure balance in these factors between the groups after weighting are described in eTables 3 and 4 in Supplement 1.

Additionally, 12 outcome-relevant covariates were submitted to a stepwise selection procedure^25^ to identify covariates for the longitudinal analyses. The stepwise procedure is a database selection approach for identifying covariates that result in better-performing models. The procedure consists of iteratively adding and removing covariates from a predictive model using a combination of a forward and backward selection approach. Akaike information criterion, Bayes information criteria, and adjusted multivariable coefficient of determination (*R*^2^ value) were used to determine the best-fitting model. This covariate selection process was conducted for each outcome at baseline, allowing for parsimonious outcome models to be tested across the 5 time points (eTable 2 in Supplement 1).

Hypotheses were tested across 24 months using mixed-effects models that compared outcomes between survivors who received DVHF and those who received SAU. Correlated random intercept and slope terms were included to allow them to vary across individuals. To account for potential selection bias, the IPWs were included as sampling weights in each mixed-effects model. To account for the fact that survivors received services from different advocates who worked within different agencies, observations were grouped by advocate and nested within each organization. Additionally, 2 variables capturing whether participants received funding and/or advocacy between 6 and 24 months were entered into the models as time-varying covariates to account for their potential influence on outcomes. Baseline levels of the outcome were included as time-invariant covariates, and whether the interview occurred before or after the onset of the COVID-19 pandemic was included as a time-varying covariate. All analyses were conducted in R,^26^ version 4.0.4, using the lme4 (version 1.1-28)^27^ and lmerTest (version 3.1-3)^28^ packages (R Project for Statistical Computing). Missing data were handled through full information maximum-likelihood estimation.

Hypothesis testing was conducted in 3 steps. First, unconditional models with time as the sole factor were estimated to examine the degree of change in the outcome across the 24-month study period. Both linear and nonlinear terms for time were examined. Second, conditional models that included intervention (0 indicating DVHF and 1 indicating SAU) and the aforementioned covariates were estimated to examine the main effects of the intervention. Finally, a time × intervention interaction term was added to the model to assess whether differences between the intervention group varied over time. These steps were repeated for each outcome. Statistical significance was set at 2-sided *P* < .05 for each step of the analysis. Effect sizes were obtained using the eff_size function included in the emmeans package,^29^ which calculated Cohen *d* using pairwise differences in the estimated means for SAU and DVHF, divided by the SD of the population.

## Results

Four hundred six individuals were interviewed shortly after they contacted one of the 5 DV agencies for services. Six months later, 375 participants (92.4%) were retained in the study. Of those retained, 30 individuals were dropped from the analytic sample due to their having received no services from the agency. One person was removed due to incomplete longitudinal data across all outcomes, resulting in a final analytic sample of 344 survivors. The sample predominantly identified as female (334 [97.1%] vs 7 male [2.0%] and 3 gender-queer or nonconforming [0.9%]) and heterosexual (299 [86.9%]). Ages ranged from 19 to 62 years (mean [SD], 34.6 [9.0] years). Most participants reported at least 1 minority racial or ethnic identity (221 [64.2%], including 36 American Indian or Alaska Native [10.5%], 16 Asian or Asian American [4.7%], 63 Black or African [18.3%], 122 Hispanic or Latinx [35.5%], 4 Middle Eastern [1.2%], and 51 as >1 race or ethnicity [14.8%] vs 123 non-Hispanic White [35.8%]), and were raising children (256 [74.4%]). Immigrant survivors included 62 participants (18.0%). The highest educational level attained varied considerably: 95 participants (27.6%) had not completed high school, 77 (22.4%) had a high school diploma or a General Educational Development certificate, 100 (29.1%) had some vocational training or had attended college classes, and 72 (20.9%) had college degrees (Table 1).

**Table 1.** Sample Descriptive Characteristics

Characteristic	Study group^a^		
	Overall analytic sample (n = 344)	DVHF model (n = 219)	SAU (n = 125)
Age at baseline, mean (SD), y	34.57 (9.05)	34.59 (9.04)	34.53 (909.80)
Gender identification			
Female	334 (97.1)	215 (98.2)	119 (95.2)
Male	7 (2.0)	2 (0.9)	5 (4.0)
Gender-queer or nonconforming	3 (0.9)	2 (0.9)	1 (0.8)
Sexual orientation			
Heterosexual	299 (86.9)	195 (89.0)	104 (83.2)
Lesbian, gay, bisexual, queer, or asexual	44 (12.8)	24 (11.0)	20 (16.0)
Race and ethnicity^b^			
American Indian or Alaska Native	36 (10.5)	21 (9.6)	15 (12.0)
Asian or Asian American	16 (4.7)	12 (5.5)	4 (3.2)
Black or African	63 (18.3)	52 (23.7)	11 (8.8)
Hispanic or Latinx	122 (35.5)	74 (33.8)	48 (38.4)
Middle Eastern	4 (1.2)	4 (1.8)	0
Non-Hispanic White	123 (35.8)	70 (32.0)	53 (42.4)
>1 Race or ethnicity	51 (14.8)	30 (13.7)	21 (16.8)
US citizenship	282 (82.0)	173 (79.0)	109 (87.2)
Primary language English	277 (80.5)	175 (79.9)	102 (81.6)
Has a disability	125 (36.3)	76 (34.7)	49 (39.2)
History of foster care	59 (17.2)	29 (13.2)	30 (24.0)
Parenting minor children	256 (74.4)	171 (78.1)	85 (68.0)
Employed in the last 6 mo	202 (58.7)	133 (60.7)	69 (55.2)
Household gross income prior year, US $			
0	20 (5.8)	10 (4.6)	10 (8.0)
<10 000	101 (29.4)	63 (28.8)	38 (30.4)
10 000-14 999	40 (11.6)	26 (11.9)	14 (11.2)
15 000-24 999	61 (17.7)	43 (19.6)	18 (14.4)
25 000-34 999	40 (11.6)	28 (12.8)	12 (9.6)
35 000-49 999	25 (7.3)	15 (6.8)	10 (8.0)
50 000-74 999	22 (6.4)	11 (5.0)	11 (8.8)
≥75 000	27 (7.8)	17 (7.8)	10 (8.0)
Educational level			
Less than high school	95 (27.6)	52 (23.7)	43 (34.4)
High school diploma or GED certificate	77 (22.4)	49 (22.4)	28 (22.4)
Vocational training or attended college classes	100 (29.1)	71 (32.4)	29 (23.2)
Associate’s, bachelor’s, or advanced degree(s)	72 (20.9)	47 (21.5)	25 (20.0)
History of homelessness	253 (73.5)	156 (71.2)	97 (77.6)
HIS score for housing instability, mean (SD)^c^			
Baseline	4.77 (1.66)	5.25 (1.55)	4.48 (1.66)
6 mo	3.38 (2.04)	2.88 (1.99)	4.26 (1.80)
12 mo	2.47 (2.11)	2.04 (1.93)	3.18 (2.22)
18 mo	2.11 (1.96)	1.85 (1.85)	2.60 (2.07)
24 mo	1.69 (1.87)	1.47 (1.72)	2.08 (2.04)
CAS score for safety, mean (SD)^d^			
Baseline	1.70 (1.13)	1.87 (1.16)	1.59 (1.09)
6 mo	0.54 (0.74)	0.47 (0.70)	0.68 (0.77)
12 mo	0.40 (0.62)	0.33 (0.54)	0.55 (0.74)
18 mo	0.34 (0.54)	0.28 (0.44)	0.44 (0.68)
24 mo	0.28 (0.5)	0.24 (0.46)	0.35 (0.55)
PHQ-9 score for depression, mean (SD)^e^			
Baseline	13.15 (6.78)	12.64 (6.18)	12.84 (6.61)
6 mo	10.21 (6.74)	9.33 (6.71)	11.74 (6.52)
12 mo	9.22 (6.78)	8.55 (6.65)	10.17 (6.88)
18 mo	8.28 (6.40)	7.62 (6.23)	9.60 (6.63)
24 mo	8.39 (6.63)	7.92 (6.73)	9.25 (6.29)
GAD-7 score for anxiety, mean (SD)^f^			
Baseline	12.31 (6.29)	13.65 (7.06)	12.1 (6.36)
6 mo	9.60 (6.35)	9.02 (6.36)	10.59 (6.18)
12 mo	8.83 (6.37)	8.31 (6.21)	9.61 (6.48)
18 mo	8.16 (6.11)	7.60 (5.96)	9.30 (6.26)
24 mo	8.13 (6.32)	7.80 (6.39)	8.76 (6.09)
TSQ score for PTSD, mean (SD)^g^			
Baseline	7.03 (2.41)	6.99 (2.43)	7.06 (2.41)
6 mo	5.89 (3.09)	5.65 (3.10)	6.27 (3.03)
12 mo	5.51 (3.13)	5.25 (3.07)	5.89 (3.17)
18 mo	5.06 (3.15)	4.82 (3.09)	5.44 (3.23)
24 mo	4.95 (3.29)	4.83 (3.26)	5.23 (3.32)

Abbreviations: CAS, Composite Abuse Scale; DVHF, Domestic Violence Housing First; GAD-7, 7-item Generalized Anxiety Disorder measure; GED, General Educational Development; HIS, 7-item Housing Instability Scale; PHQ-9, 9-item Patient Health Questionnaire; PTSD, posttraumatic stress disorder; SAU, services as usual; TSQ, 10-item Trauma Screening Questionnaire.

^a^ Descriptive characteristics are based on unweighted observations. Unless otherwise indicated, data are expressed as No. (%) of participants. Owing to missing data, percentages may not total 100.

^b^ Participants could choose more than 1 race or ethnicity.

^c^ Scores ranged from 0 to 7, with higher scores indicating greater housing instability.

^d^ Scores ranged from 0, indicating never, to 5, indicating daily.

^e^ Scores ranged between 0 and 27, with higher scores indicating higher depression.

^f^ Scores ranged between 0 and 21, with higher scores indicating higher anxiety.

^g^ Scores could range from 0 to 10, with higher scores indicating a higher degree of PTSD.

**Figure 1.**
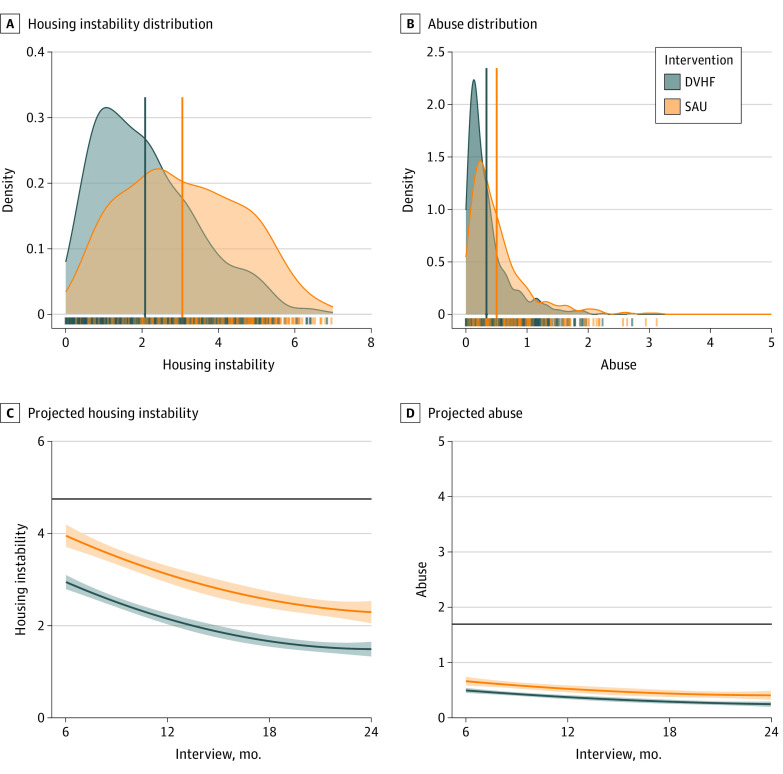
Housing Instability and Total Abuse by Intervention A and B, Distributions and means (solid vertical lines) for the estimated outcomes (housing instability and abuse) were stratified by intervention (Domestic Violence Housing First [DVHF] model and services as usual [SAU]). Numeric data are given in Table 2, under the conditional main effect model. C and D, Estimated outcomes and 95% CI bands for DVHF and SAU by interview. The solid black lines represent the overall mean for each outcome at baseline. Estimates are based on weighted observations.

Housing instability significantly decreased over 2 years (linear β = −0.75 [95% CI, −0.94 to −0.56; *P* < .001]; quadratic β = 0.46 [95% CI, 0.27-0.65; *P* < .001]) (Table 2). The results of the conditional main effects model indicated an association between the intervention and housing stability, with those who received SAU experiencing more housing instability than those who received the DVHF model (mean difference, 0.78 [95% CI, 0.42-1.14]; β = 0.37 [95% CI, 0.21-0.53]; *P* < .001), with a medium effect size (Cohen *d* = 0.62 [95% CI, 0.33-0.91]) (Figure 1A). The interaction between time and intervention was not significant (Figure 1C), indicating that the group differences between those who received DVHF or SAU did not vary significantly across the study period.

**Table 2.** Mixed-Effects Models Estimating Housing Instability and Domestic Violence Among 344 Study Participants

	Unconditional model		Conditional main effects model		
	β (95% CI)	*P* value	Estimated mean difference (95% CI)	β (95% CI)	*P* value
**Housing instability**					
Intercept	−0.08 (−0.27 to 0.12)	.44	NA	−0.46 (−0.95 to 0.03)	.07
Linear time	−0.75 (−0.94 to −0.56)	<.001	NA	−0.52 (−0.74 to −0.31)	<.001
Quadratic time	0.46 (0.27 to 0.65)	<.001	NA	0.27 (0.06 to 0.47)	.01
Intervention^a^	NA	NA	0.78 (0.42 to 1.14)	0.37 (0.21 to 0.53)	<.001
**Domestic violence**					
Intercept	−0.03 (−0.12 to 0.05)	.43	NA	−0.30 (−0.58 to −0.03)	.03
Linear time	−0.39 (−0.60 to −0.19)	<.001	NA	−0.20 (−0.43 to 0.03)	.10
Quadratic time	0.24 (0.04 to 0.44)	.02	NA	0.12 (−0.11 to 0.34)	.31
Intervention^a^	NA	NA	0.15 (0.05 to 0.26)	0.24 (0.09 to 0.40)	.002

Abbreviation: NA, not applicable.

^a^ Reference group consists of survivors who received the Domestic Violence Housing First (DVHF) model (0 indicates DVHF; 1 indicates services as usual).

The IPV significantly decreased over 2 years (linear β = −0.39 [95% CI, −0.60 to −0.19; *P* < .001]; quadratic β = 0.24 [95% CI, 0.04-0.44; *P* = .02]) (Table 2). The results of the conditional main effects model indicated an association between the intervention and domestic abuse, with those who received SAU experiencing greater abuse over 2 years compared with those who received the DVHF model (mean difference, 0.15 [95% CI, 0.05-0.26]; β = 0.24 [95% CI, 0.09-0.40]; *P* = .002), with a small effect size (Cohen *d* = 0.25 [95% CI, 0.01-0.49]) (Figure 1B). The interaction between time and intervention was not significant (Figure 1D), indicating that the group differences between those who received DVHF or SAU did not vary significantly across the study period.

**Figure 2.**
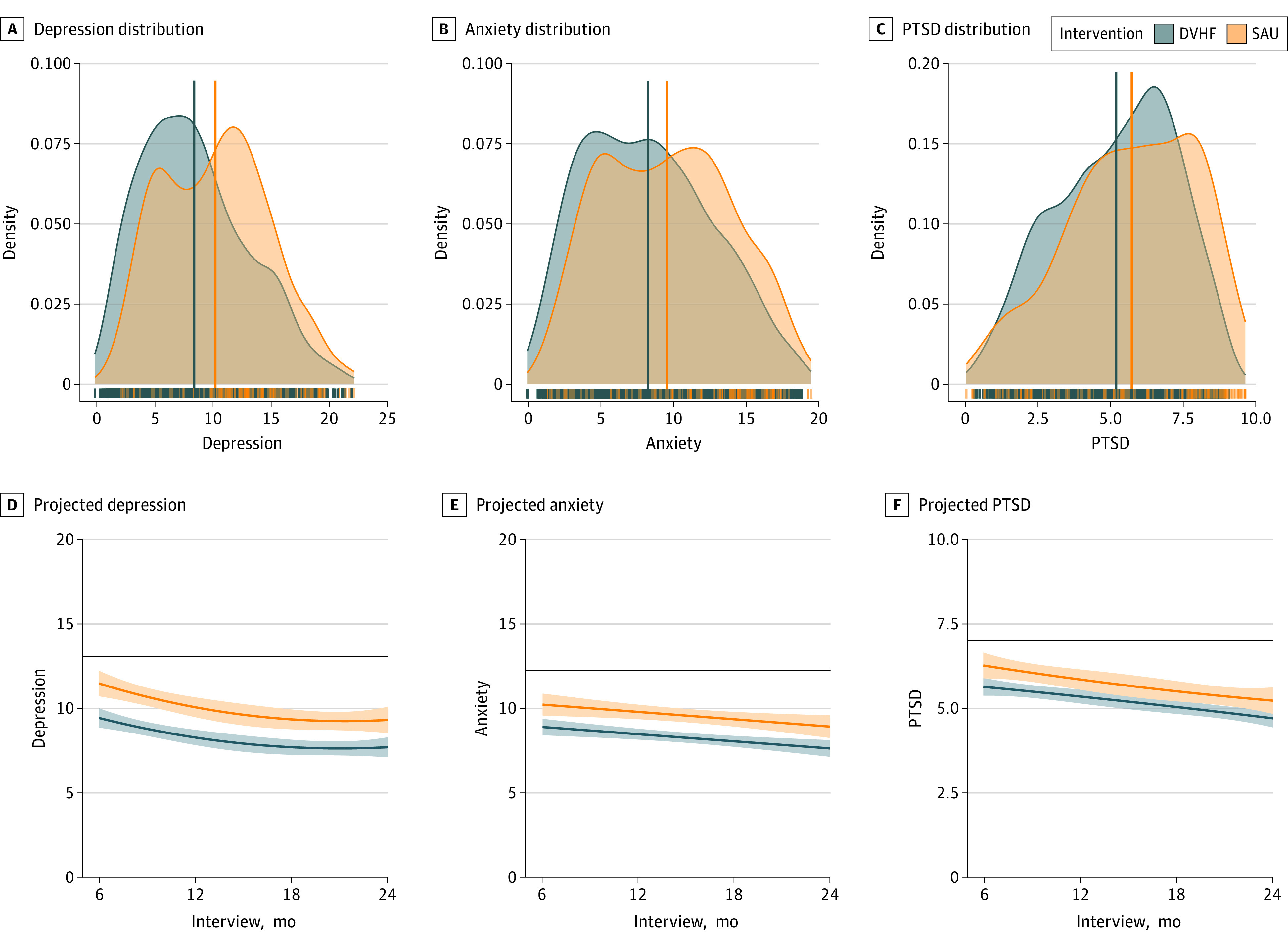
Depression, Anxiety, and Posttraumatic Stress Disorder (PTSD) by Intervention A through C, Distributions and means (solid vertical lines) for the estimated outcomes (depression, anxiety, and PTSD) were stratified by intervention (Domestic Violence Housing First [DVHF] model and service as usual [SAU]). The y-axis density values represent the probability of the data distribution occurring within the range determined by the x-axis. The area under the density curve represents probability and therefore equals to 1. The rug plot displays marginal distributions of single data points. Numeric data are given in Table 3, under the conditional main effects model. D through F, Estimated outcomes and 95% CI bands for DVHF and SAU by interview. The solid black lines represent the overall mean score for each outcome at baseline. Estimates are based on weighted observations.

Depression significantly decreased over 2 years (linear β = −0.40 [95% CI, −0.60 to −0.20; *P* < .001]; quadratic β = 0.30 [95% CI, 0.10-0.51; *P* = .003]) (Table 3). The results of the conditional main effects model indicated an association between the intervention and depression, with those who received SAU experiencing more depression over 2 years compared to those who received the DVHF model (mean difference, 1.35 [95% CI, 0.27-2.43]; β = 0.20 [95% CI, 0.05-0.35]; *P* = .008), with a small effect size (Cohen *d* = −0.32 [95% CI, −0.58 to −0.06]) (Figure 2A). The interaction between time and intervention was not significant (Figure 2D), indicating that the group differences between those who received DVHF or SAU did not vary significantly across the study period.

**Table 3.** Mixed-Effects Models Estimating Depression, Anxiety, and PTSD Among 344 Study Participants

	Unconditional model		Conditional main effects model		
	β (95% CI)	*P* value	Estimated mean difference (95% CI)	β (95% CI)	*P* value
**Depression**					
Intercept	−0.05 (−0.15 to 0.05)	.36	NA	−0.05 (−0.39 to 0.29)	.77
Linear time	−0.40 (−0.60 to −0.20)	<.001	NA	−0.38 (−0.61 to −0.15)	.001
Quadratic time	0.30 (0.10 to 0.51)	.003	NA	0.31 (0.08 to 0.53)	.01
Intervention^a^	NA	NA	1.35 (0.27 to 2.43)	0.20 (0.05 to 0.35)	.008
**Anxiety**					
Intercept	−0.03 (−0.13 to 0.07)	.53	NA	0.001 (−0.35 to 0.35)	>.99
Linear time	−0.07 (−0.12 to −0.03)	<.001	NA	−0.02 (−0.08 to 0.05)	.57
Intervention^a^	NA	NA	1.15 (0.11 to 2.19)	0.18 (0.03 to 0.34)	.02
**PTSD**					
Intercept	−0.04 (−0.12 to 0.05)	.41	NA	−0.10 (−0.51 to 0.32)	.65
Linear time	−0.11 (−0.15 to −0.07)	<.001	NA	−0.09 (−0.15 to −0.02)	.01
Intervention^a^	NA	NA	0.54 (0.04 to 1.04)	0.17 (0.02 to 0.32)	.02

Abbreviations: NA, not applicable; PTSD, posttraumatic stress disorder.

^a^ Reference group consists of survivors who received the Domestic Violence Housing First (DVHF) model (0 indicates DVHF; 1 indicates services as usual).

Anxiety significantly decreased over 2 years (linear β = −0.07 [95% CI, −0.12 to −0.03]; *P* < .001) (Table 3). The results of the conditional main effects model indicated an association between the intervention and anxiety, with those who received SAU experiencing higher anxiety over 2 years compared with those who received the DVHF model (mean difference, 1.15 [95% CI, 0.11-2.19]; β = 0.18 [95% CI, 0.03-0.34]; *P* = .02), with a small effect size (Cohen *d* = −0.29 [95% CI, −0.55 to −0.02]) (Figure 2B). The interaction between time and intervention was not significant (Figure 2E), indicating the group differences between those who received DVHF or SAU did not vary significantly across the study period.

Posttraumatic stress disorder significantly decreased over 2 years (linear β = −0.11 [95% CI, −0.15 to −0.07]; *P* < .001) (Table 3). The results of the conditional main-effects model indicated an association between the intervention and PTSD, with those who received SAU experiencing higher PTSD over 2 years compared with those who received the DVHF model (mean difference, 0.54 [95% CI, 0.04-1.04]; β = 0.17 [95% CI, 0.02-0.32]; *P* = .02), with a small effect size (Cohen *d* = −0.25 [95% CI, −0.49 to −0.01]) (Figure 2C). The interaction between time and intervention was not significant (Figure 2F), indicating the group differences between those who received DVHF or SAU did not vary significantly across the study period.

## Discussion

In this comparative effectiveness study, survivors who received the DVHF intervention experienced improved housing stability, safety, and mental health over 2 years compared with those who received SAU. Results corroborated findings from a pilot study of the DVHF model^30^ as well as analyses of the current study’s data across 6 and 12 months.^27,31^ It is promising that DVHF resulted in relatively rapid improvements for survivors (6 months after they sought services) and that these positive changes were maintained across 12, 18, and 24 months.

It should be noted that housing stability, safety, and mental health improved for the sample overall, with those receiving the DVHF intervention experiencing even greater positive outcomes. While we cannot categorically conclude that the positive changes experienced by those receiving SAU were due to services received, given that the sample did not include survivors who received no services, the positive change likely speaks to the value of DV services overall, which are designed to promote safety and well-being through survivor-driven services.^32,33,34^ While the model is congruent with the mission of DV agencies to be survivor driven and trauma informed,^32,33,35^ agencies are currently limited in their ability to offer the DVHF model due to lack of funding for both intensive advocacy and flexible funding. This work can be time-consuming and complex.^36^ Funding is needed for staff training and to flexibly meet the diverse needs of survivors. At least some of these funds may need to come from private foundations and community members rather than from governmental sources, given tighter restrictions on the use of taxpayer dollars. However, given the incredible societal cost-burden due to IPV, homelessness, and mental health disorders,^37,38^ funding a model that reduces all of these social ills would be a prudent investment in public health.

### Research and Policy Implications

This is the first study, to our knowledge, to longitudinally evaluate the DVHF model, and more studies are needed to build on these findings. Future research could examine the unique contribution of either survivor-driven housing-inclusive advocacy or flexible funding to survivors’ life circumstances and well-being over time. However, we caution against attempting to determine a “one size fits all” intervention. It is critical that services provided to survivors continue to be individualized to each survivor’s needs and circumstances.^32,33,34^ Some survivors request only brief or crisis services while others seek long-term help. Any studies examining the relative importance of advocacy vs funding should bear in mind that survivors enter services with unique needs.

### Limitations

Results need to be considered in light of study limitations. Practical and ethical considerations led us to choose a nonrandomized comparative effectiveness study design; therefore, participants were not randomly assigned to the DVHF or SAU groups. We judiciously ensured the accuracy of grouping participants by services received and controlled for preexisting group differences. However, there may be unidentified associations that contributed to which services participants received or that may have accounted for outcomes achieved.

Most study participants identified as cisgender heterosexual women; thus, it is unclear how generalizable these findings are to those who identify as heterosexual men or LGBTQ (lesbian, gay, bisexual, transgender, or queer) individuals. Further, while the study was racially and ethnically diverse, few participants were American Indian or Alaska Native or of Asian or Middle Eastern descent. The study setting included both urban and rural areas but was located in the Pacific Northwest. Geographical differences, varying state and local laws and policies, and housing availability may change the effectiveness of this model. Furthermore, this study did not include survivors who received no services from DV agencies.

Significant differences were found between those who received the DVHF intervention and those who received SAU, which suggested that those in the DVHF intervention had fewer barriers and greater assets at baseline compared with those who received SAU. While these differences were accounted for statistically using IPWs, these findings may indicate selection bias on the part of staff. Future studies should continue identifying ways of optimizing equitable intervention selection processes.^39^

## Conclusions

Intimate partner violence, housing instability, and mental health disorders are individually and collectively public health concerns of sizable magnitude. Prior research has demonstrated the interrelationships among them as well as how they can reinforce each other.^40,41^ The DVHF model’s amelioration of all these interconnected public health issues—relatively quickly and with long-term continuance—will be of substantial interest to DV agencies and others working to support unstably housed IPV survivors.

## Notes

Supplement 1. **eMethods.** Inverse Probability Weighting and Balance Diagnostics **eTable 1.** Logistic Regressions With Intervention (SAU or DVHF) Associated With Baseline Characteristics **eTable 2.** Covariates Included in Each Mixed-Effects Model, by Outcome **eTable 3.** Diagnostic Results for Inverse Probability Weighting: Overidentification Test for Covariate Balance **eTable 4.** Diagnostic Results for Inverse Probability Weighting: Balance of Covariates
eReferences

Supplement 2.
Data Sharing Statement
