# Supplementary material for: Domestic Violence Housing First Model and Association With Survivors’ Housing Stability, Safety, and Well-being Over 2 Years
Source: JAMA Netw Open. 2023 Jun 26;6(6):e2320213. doi: 10.1001/jamanetworkopen.2023.20213 (PMC10293912; doi:10.1001/jamanetworkopen.2023.20213)
Supplement: Supplement 1. — eMethods. Inverse Probability Weighting and Balance Diagnostics eTable 1. Logistic Regressions With Intervention (SAU or DVHF) Associated With Baseline Characteristics eTable 2. Covariates Included in Each Mixed-Effects Model, by Outcome eTable 3. Diagnostic Results for Inverse Probability Weighting: Overidentification Test for Covariate Balance eTable 4. Diagnostic Results for Inverse Probability Weighting: Balance of Covariates eReferences [file jamanetwopen-e2320213-s001.pdf]

Supplementary Online Content

Sullivan CM, Simmons C, Guerrero M, et al. Domestic Violence Housing First model and association with survivors' housing stability, safety, and well-being over 2 years. *JAMA Netw Open*. 2023;6(6):e2320213. doi:10.1001/jamanetworkopen.2023.20213

eMethods. Inverse Probability Weighting and Balance Diagnostics

eTable 1. Logistic Regressions With Intervention (SAU or DVHF) Associated With Baseline Characteristics

eTable 2. Covariates Included in Each Mixed-Effects Model, by Outcome

eTable 3. Diagnostic Results for Inverse Probability Weighting: Overidentification Test for Covariate Balance

eTable 4. Diagnostic Results for Inverse Probability Weighting: Balance of Covariates

eReferences

This supplementary material has been provided by the authors to give readers additional information about their work.

© 2023 Sullivan CM et al. *JAMA Network Open*.

eMethods. Inverse Probability Weighting and Balance Diagnostics

Inverse probability weights[1] were included in the mixed effect models sampling weights to account for selection bias resulting from factors that increased the probability that certain individuals received the DVHF model. Prior to estimating the IPWs, we first used logistic regression models to examine whether there were any meaningful baseline differences between those who received DVHF versus those receiving SAU across 72 variables (Table S1). Statistical significance was set at $p < .05$. Thirteen factors were identified and used to calculate the IPWs: parenting children (y/n), living with the abuser (y/n), racial/ethnic minority (y/n), having been in foster care as a child (y/n), housing barriers, housing instability, staying with friends to avoid homelessness (y/n), inability to make ends meet, overall abuse, alcohol misuse, drug misuse, quality of life, and whether or not the DV agency was in a rural area (y/n). Next, a propensity score ($p(x)=P(T=1|X=x)$), or the probability of treatment based on relevant measured baseline covariates, was obtained for each individual. The inverse of the propensity score ($w(x)=1/p(x)$ for treated individuals and $w(x)=1/(1-p(x))$ for untreated individuals) was used as a weight.

To ensure that our groups were balanced (i.e. there are no systematic differences in the baseline factors between the DVHF and SAU groups),[2,3] we conducted an overidentification test for covariate balance[4], which tests whether the distribution of a covariate is the same for all treatment levels (Table S3), and compared the standardized differences and variance ratios between DVHF and SAU in the raw and weighted data (Table S4).

© 2023 Sullivan CM et al. *JAMA Network Open*.

eTable 1. Logistic Regressions With Intervention (SAU or DVHF) Associated With Baseline Characteristics

Variable		Odds Ratio	SE	*p*	95% CI	
1.	Age	0.999	0.012	0.947	0.975	1.024
2.	Hispanic/Latinx (Y/N)	0.828	0.192	0.416	0.526	1.304
**3.**	**Racial Minority (Y/N)**	**1.616**	**0.372**	**0.037**	**1.029**	**2.538**
4.	English as primary language (Y/N)	1.137	0.323	0.652	0.651	1.985
5.	Cisgender (Y/N)	0.959	0.235	0.866	0.593	1.551
6.	Heterosexual (Y/N)	1.502	0.485	0.208	0.797	2.830
7.	US citizen (Y/N)	0.543	0.170	0.052	0.294	1.004
8.	Involved with abuser (Y/N)	0.498	0.183	0.058	0.242	1.023
9.	Homeless as child	0.872	0.235	0.612	0.514	1.479
10.	In agency shelter (Y/N)	0.911	0.207	0.683	0.584	1.423
**11.**	**Living with abuser (Y/N)**	**0.410**	**0.179**	**0.041**	**0.174**	**0.964**
12.	Length of relationship with abuser (in months)	0.999	0.001	0.347	0.996	1.001
13.	Length of abuse (in days)	1.000	0.000	0.826	1.000	1.000
14.	Overall physical health	1.125	0.119	0.268	0.913	1.385
**15.**	**Children (Y/N)**	**1.703**	**0.424**	**0.033**	**1.045**	**2.775**
16.	Number of children	0.993	0.083	0.930	0.843	1.170
17.	Use of child	1.095	0.127	0.435	0.872	1.376
18.	Employed in last 6-months	1.299	0.292	0.244	0.836	2.018
19.	Feelings about employment	1.036	0.059	0.536	0.927	1.158
20.	Enrolled in school	1.825	0.588	0.062	0.970	3.433
21.	Access to car (Y/N)	0.969	0.225	0.891	0.615	1.526
22.	Driver's license (Y/N)	1.464	0.337	0.098	0.932	2.299
23.	Less than high school education (Y/N)	1.082	0.051	0.096	0.986	1.187

© 2023 Sullivan CM et al. *JAMA Network Open*.

24. Depression	0.986	0.016	0.384	0.955	1.018
25. Anxiety	0.988	0.017	0.480	0.954	1.022
26. PTSD	1.020	0.046	0.665	0.933	1.115
27. Difficulty paying bills	0.972	0.160	0.865	0.704	1.344
28. Borrowed money for rent or mortgage	1.240	0.282	0.343	0.795	1.936
29. Lifetime homelessness	1.000	0.000	0.374	1.000	1.000
**30. Foster care (Y/N)**	**0.500**	**0.143**	**0.016**	**0.285**	**0.877**
31. Housing barriers	0.570	0.148	0.031	0.343	0.948
**32. Stayed with friends or family to avoid homelessness (as an adult)**	**0.412**	**0.161**	**0.024**	**0.191**	**0.888**
**33. Inability to make ends meet**	**0.849**	**0.061**	**0.023**	**0.737**	**0.977**
34. Financial strain	0.980	0.092	0.828	0.814	1.179
35. Physical disability (Y/N)	0.812	0.186	0.362	0.519	1.271
36. Mental health issues (Y/N)	1.033	0.248	0.893	0.646	1.652
**37. Overall abuse**	**0.699**	**0.106**	**0.019**	**0.519**	**0.943**
38. Economic abuse - restriction of finances	0.881	0.077	0.149	0.742	1.046
39. Economic abuse - financial exploitation	0.898	0.097	0.319	0.726	1.110
**40. Drug misuse**	**0.781**	**0.074**	**0.009**	**0.649**	**0.940**
**41. Alcohol misuse**	**0.779**	**0.091**	**0.032**	**0.620**	**0.978**
42. Internal tools related to safety	1.322	0.252	0.143	0.910	1.922
43. Trade-offs related to safety	1.207	0.151	0.134	0.944	1.543
44. Expectations of support related to safety	1.117	0.164	0.450	0.838	1.490
45. Hope	1.462	0.320	0.083	0.952	2.246
46. Positive emotions	1.223	0.143	0.086	0.972	1.539
47. Negative emotions	0.833	0.093	0.102	0.670	1.037
48. Social support	0.844	0.083	0.084	0.697	1.023
**49. Quality of life**	**1.268**	**0.123**	**0.015**	**1.048**	**1.535**

© 2023 Sullivan CM et al. *JAMA Network Open*.

**50. Seeking help with housing**	**0.119**	**0.124**	**0.041**	**0.015**	**0.916**
51. Seeking help with employment	0.945	0.214	0.803	0.607	1.473
52. Seeking help with education	0.880	0.205	0.584	0.557	1.390
53. Seeking help with finances	1.858	0.735	0.117	0.856	4.035
54. Seeking legal help	0.728	0.184	0.209	0.444	1.195
55. Seeking help with childcare	1.138	0.257	0.566	0.731	1.772
56. Seeking help with counseling	0.864	0.277	0.647	0.461	1.618
57. Seeking help w transportation	1.420	0.318	0.117	0.916	2.202
58. Seeking help with healthcare	0.879	0.203	0.577	0.560	1.382
59. Seeking help children's needs	1.061	0.239	0.791	0.683	1.650
60. Seeking help with food	0.722	0.168	0.162	0.458	1.139
61. Seeking help with clothing	0.712	0.169	0.153	0.446	1.134
62. Seeking help for material goods	1.101	0.265	0.690	0.687	1.764
63. Seeking help with social support	1.213	0.365	0.522	0.672	2.187
**64. Housing Instability**	**0.713**	**0.054**	**0.000**	**0.616**	**0.827**
65. Sexual Abuse	0.923	0.068	0.273	0.799	1.065
**66. Stalking**	**0.843**	**0.058**	**0.014**	**0.736**	**0.966**
67. Physical Abuse	0.861	0.087	0.140	0.706	1.050
68. Emotional Abuse	0.850	0.073	0.058	0.719	1.006
69. Economic Abuse	0.866	0.092	0.173	0.703	1.065
**70. Rural/Urban**	**0.391**	**0.091**	**0.000**	**0.248**	**0.618**
71. Reads English	1.071	0.160	0.646	0.799	1.437
72. Household Income	0.996	0.047	0.935	0.909	1.092

Note. Two factors were omitted: "Seeking help with housing" perfectly predicted cases, which would have resulted in their exclusion from the model; and "Stalking" is a subscale of Overall Abuse (which was included in the model) and the two baseline scores were highly correlated ($r = 0.811$). Reference group is survivors who received services as usual (0= SAU, 1= DVHF).

© 2023 Sullivan CM et al. *JAMA Network Open*.

eTable 2. Covariates Included in Each Mixed-Effects Model, by Outcome

Housing Instability	Domestic Violence	
1. Financial difficulty	Financial difficulty	
2. US citizenship (Y/N)	Have children (Y/N)	
3. Employment in last 6 months (Y/N)	Age	
4. Lifetime homelessness in days	US citizenship	
5. Have children (Y/N)		
6. Relationship with abuser (Y/N)		

Depression	Anxiety	PTSD
1. Have a disability (Y/N)	Financial difficulty	Have disability (Y/N)
2. Less than high school education (Y/N)	Racial and ethnic minoritized identity (Y/N)	Have children (Y/N)
3. Financial difficulty	Have a disability (Y/N)	Financial difficulty
4. Have children (Y/N)	Have children (Y/N)	Read English (Y/N)
5. Relationship with abuser (Y/N)		Less than high school education (Y/N)
6. US citizenship		Age

© 2023 Sullivan CM et al. *JAMA Network Open*.

eTable 3. Diagnostic Results for Inverse Probability Weighting: Overidentification Test for Covariate Balance

$X^2(14)$	8.19
p-value	0.88

Note. The null hypothesis that the IPW model is balanced was not rejected, indicating that the covariates are balanced between the intervention groups.

© 2023 Sullivan CM et al. *JAMA Network Open*.

eTable 4. Diagnostic Results for Inverse Probability Weighting: Balance of Covariates

	Standardized Differences		Variance Ratio	
	Raw	Weighted	Raw	Weighted
Parenting children (Y/N)	0.23	-0.01	0.79	1.01
Living with abuser	-0.23	-0.02	0.46	0.93
Racial/ethnic minority	0.24	-0.03	0.87	1.02
Foster Care	-0.29	0.00	0.61	1.00
Housing Barriers	-0.25	0.01	1.30	0.99
Housing Instability	-0.51	0.01	1.16	0.84
Staying with friends to avoid homelessness	-0.25	-0.08	1.87	1.24
Inability to make ends meet	-0.22	0.08	1.26	0.88
Overall abuse	-0.28	-0.04	0.82	1.23
Alcohol misuse	-0.20	-0.01	0.48	0.79
Drug misuse	-0.27	0.04	0.44	0.76
Quality of Life	0.31	0.00	1.07	1.11
Rural/Urban	-0.47	-0.04	1.17	1.01

© 2023 Sullivan CM et al. *JAMA Network Open*.

eReferences

1. Hernán MA, Robins JM. Causal inference. CRC Boca Raton, FL; 2010.
2. Austin PC, Stuart EA. Moving towards best practice when using inverse probability of treatment weighting (IPTW) using the propensity score to estimate causal treatment effects in observational studies. *Stat Med*. Dec 10 2015;34(28):3661-79. doi:10.1002/sim.6607
3. Austin PC. Balance diagnostics for comparing the distribution of baseline covariates between treatment groups in propensity-score matched samples. *Stat Med*. Nov 10 2009;28(25):3083-107. doi:10.1002/sim.3697
4. Imai K, Ratkovic M. Covariate balancing propensity score. *Journal of the Royal Statistical Society: Series B (Statistical Methodology)*. 2014;76:243-263. doi:10.1111/rssb.12027

© 2023 Sullivan CM et al. *JAMA Network Open*.
