# Supplementary material for: Domestic Violence Housing First Model and Association With Survivors’ Housing Stability, Safety, and Well-being Over 2 Years
Source: JAMA Netw Open. 2023 Jun 26;6(6):e2320213. doi: 10.1001/jamanetworkopen.2023.20213 (PMC10293912; doi:10.1001/jamanetworkopen.2023.20213)
Supplement: Supplement 2. — Data Sharing Statement [file jamanetwopen-e2320213-s002.pdf]

Data Sharing Statement

Sullivan. Domestic Violence Housing First Model and Association With Survivors' Housing Stability, Safety, and Well-being Over 2 Years. *JAMA Netw Open*. Published June 26, 2023. doi:10.1001/jamanetworkopen.2023.20213

Data
Data available: Yes
Data types: Deidentified participant data
How to access data: Request from Dr. Cris Sullivan: sulliv22@msu.edu
When available: With publication

Supporting Documents
Document types: None

Additional Information
Who can access the data: Researchers whose proposed use of the data has been approved
Types of analyses: For any purpose
Mechanisms of data availability: With a signed data access agreement
